# A genome-scale metabolic model of potato late blight suggests a photosynthesis suppression mechanism

**DOI:** 10.1186/s12864-018-5192-x

**Published:** 2018-12-11

**Authors:** Kelly Botero, Silvia Restrepo, Andres Pinzón

**Affiliations:** 1 Grupo de Bioinformática y Biología de Sistemas, Universidad Nacional del Colombia - Instituto de Genética, Calle 53- Carrera 32, Edificio 426, Bogotá, Colombia; 2 Laboratorio de Micología y Fitopatología, Universidad de los Andes, Bogotá, Colombia; 3 Centro de Bioinformática y Biología Computacional, Manizales, Colombia

**Keywords:** Compatible interaction, *Phytophthora infestans*, *Solanum tuberosum*, Systems biology, Metabolic reconstruction, Flux balance analysis

## Abstract

**Background:**

*Phytophthora infestans* is a plant pathogen that causes an important plant disease known as late blight in potato plants (*Solanum tuberosum*) and several other solanaceous hosts. This disease is the main factor affecting potato crop production worldwide. In spite of the importance of the disease, the molecular mechanisms underlying the compatibility between the pathogen and its hosts are still unknown.

**Results:**

To explain the metabolic response of late blight, specifically photosynthesis inhibition in infected plants, we reconstructed a genome-scale metabolic network of the *S. tuberosum* leaf, PstM1. This metabolic network simulates the effect of this disease in the leaf metabolism. PstM1 accounts for 2751 genes, 1113 metabolic functions, 1773 gene-protein-reaction associations and 1938 metabolites involved in 2072 reactions. The optimization of the model for biomass synthesis maximization in three infection time points suggested a suppression of the photosynthetic capacity related to the decrease of metabolic flux in light reactions and carbon fixation reactions. In addition, a variation pattern in the flux of carboxylation to oxygenation reactions catalyzed by RuBisCO was also identified, likely to be associated to a defense response in the compatible interaction between *P. infestans* and *S. tuberosum*.

**Conclusions:**

In this work, we introduced simultaneously the first metabolic network of *S. tuberosum* and the first genome-scale metabolic model of the compatible interaction of a plant with *P. infestans*.

**Electronic supplementary material:**

The online version of this article (10.1186/s12864-018-5192-x) contains supplementary material, which is available to authorized users.

## Background

Plants use various strategies to resist pathogen attack and to avoid the development of diseases, such as, massive transcriptional reprogramming, production of reactive oxygen species (ROS), reinforcements of the cell wall and synthesis of antimicrobial secondary metabolites and pathogenesis-related proteins involved in resistance [1–3]. In those cases when plants resist pathogen attack, plants they are considered *resistant* to that specific disease, and pathogens are referred to as avirulent on that plant. This type of plant-pathogen interaction is thus known as incompatible. On the other hand, sometimes plant pathogens develop strategies to evade plant defense responses, become virulent and establish the disease. In those cases, the plant-pathogen interaction is known as *compatible* and the infected plant is considered *non-*resistant or susceptible [4, 5]. To some plant pathogens, these evasion mechanisms are, at least, partially known [1, 6], while to some others, these mechanisms remain unknown. This latter is the case of *Phytophthora infestans*, one of the most destructive pathogens of *Solanum tuberosum*. This susceptibility is triggered by effector proteins of the pathogen [1], some of which are inhibitors that target defense-related proteins, and processes such as programmed cell death in plants [7].

*P. infestans* is a hemi-biotrophic plant pathogen causing the disease known as late blight of potato, which was the same plant disease responsible for the Irish famine in the mid-nineteenth century [8]. *S. tuberosum* is the fourth leading food crop (after corn, rice, and wheat) for human consumption [9], and it is one of the most produced crops worldwide. Historically, potato late blight is the main factor affecting potato crop production [10, 11]. After its first pandemic in the middle of the nineteenth century, *P. infestans* has remained as the most destructive pathogen in plantations of this crop, leading to annual losses that would have been enough to feed several hundred million people [12]. The economic value of this loss and the cost of crop protection are estimated at 6.7 billion dollars a year [13].

Successful biological systems analysis requires that we understand the functional interactions between the key components of biological organisms and how these interactions change in disease states. Computational modeling has been widely used in order to understand the complexity that arises from the understanding of such interactions, by means of the application of mathematics, physics and computer science. Computational models allow us to understand how new properties emerge from the interconnection between system components and thus to study their behavior in response to stimuli and changes in the environment [14]. Even though computational modeling of potato late blight has been studied since the early 1950s, it was not until a few years ago that important molecular information to feed these models was accessible to the research community. Typically, this information has been gathered by heterogeneous approaches, which have covered several research fields such as structural and comparative genomics, protein-protein interactions and differential gene expression [15]. In particular, in the field of gene expression, several investigations have been carried out using microarray based assays on cDNA clones [16], transcriptome analysis through DeepSAGE [17] and RNA-Seq analysis [18].

These approaches have generated large amounts of data, allowing us to understand, to a certain extent, some of the main mechanisms involved in potato late blight. Nevertheless, due to the heterogeneity of this information, a full view of the disease is still fragmented. For this reason, and to get a better understanding of the molecular mechanisms underlying the compatible interaction inside the host cell, it is highly suitable and necessary to gather and integrate this information into a single computational model of the disease. This model should capture the time-dependent nature of this biological system, integrate various ranges of spatial and temporal scales, and allow us to explore the possible molecular mechanisms underlying the compatible interaction.

To this end, in recent years, the scientific community has made multiple efforts to integrate functional genomic characterization and biochemical knowledge into models known as genomic-scale metabolic reconstructions (GEMR). These models are based on a detailed knowledge of the system’s individual components (functional annotation), to allow the reconstruction of the system through a bottom-up approach. The aim is to understand the properties that arise from the interactions between the metabolic system’s components in response to environmental stimuli, and their behavior through time [19–22].

During the past years, this approach has been widely implemented in plants, with the development of GEMRs for several species, including *Arabidopsis thaliana* [23–26], *Hordeum vulgare* [27], *Zea mays* [28–30], *Sorghum bicolor* [28], *Saccharum officinarum* [28]; *Brassica napus* [31, 32], *Oryza sativa* [33], and *Solanum lycopersicum* [34]. In general, the development of these models can be synthesized into four fundamental steps: 1) Automatic reconstruction based on a genome annotation and biochemical databases. 2) Manual refinement of the reconstruction through a literature review with the aid of biochemical and metabolic databases. In this step, each gene and each reaction are verified, so that they are correctly located and connected. 3) Mathematical and computational formalization of the biochemical information, where the system’s specific conditions and limits are defined. This model is validated through multiple iterations and is used to prospectively simulate the system’s phenotypic behavior. 4) Verification, evaluation, and validation of the reconstruction. In other words, if we want, for example, to simulate the production of biomass precursors in an organism, during this step the reliability of the model to correctly portrait this is then evaluated.. Often, this evaluation leads to the identification of incorrect metabolic functions in the reconstruction (network gaps), which are once again evaluated by steps 2 and 3. Therefore, the whole process is iterative and the model is generally susceptible to being continually refined [35].

Once the mathematical model is generated in step 3, the phenotypic predictions of the organism’s metabolic activity can be established through modelling methods based on restrictions, which include an in silico simulation of the model under inferred metabolic objectives and a set of restrictions that represent genetic or environmental conditions [36, 37]. A widely used method is flux balance analysis (FBA), which performs the linear optimization of a metabolic network in steady-state, in order to predict an optimal set of flux values that are coherent with the maximization or minimization of the chosen objective (which depends on the purpose of the study) and under restrictions imposed on the reaction fluxes [38].

Given that genomic information used to generate the metabolic reconstructions does not consider the real expression of each gene or the subcellular localization of gene products, flux restrictions based on different “omics” data (such as transcriptomics, proteomics, and metabolomics) must be integrated into the GEMRs, in order to recreate specific metabolic phenotypes [30, 39, 40]. This approach has a powerful scope for gaining knowledge of the molecular and biochemical mechanisms of plants under specific environmental or genetic conditions. In addition, this approach allow us to contextualize high-throughput data, as well as to guide hypothesis-driven discovery or to identify novel network properties [21].

In this study, we report the first genome-scale reconstruction of *S. tuberosum* and generate a genome-scale metabolic model of the compatible interaction between *S. tuberosum* and *P. infestans*, through the incorporation of expression data of the pathosystem into the model. Previous work on the compatible interaction had already identified a decrease in photosynthetic activity of infected plants, however, the underpinnings behind it remain unknown [16]. Hence, this model was aimed to follow up on those findings and to try to understand the molecular bases of this typical photosynthesis turn off during plant disease. Furthermore, the model will be useful for understanding other molecular mechanisms involved during the pathosystem activity and for proposing novel directed hypotheses, guiding research to conduct metabolic engineering in the plant, and identifying emerging properties of the compatible interaction, which otherwise could not be observed through the study of individual molecules and processes.

## Results and discussion

The reconstruction of the genome-scale metabolic model followed six major steps: (1) automatic reconstruction of draft network via homology searches for the identification of metabolic activities and biochemical reactions; (2) manual and semiautomatic refinement of the reconstruction (3) establishment of gene-protein-reaction (GPR) association; (4) generation of a genome-scale metabolic model in steady state; (5) incorporation of gene expression data of the compatible interaction into the metabolic model; (6) flux balance analysis using a pre-defined objective function. In addition, model predictions were contrasted against experimental observations.

### Metabolic reconstruction

The draft network included 1288 reactions and 1482 metabolites, 217 of which were dead-ends that decreased the metabolic network’s connectivity. A manual search of the dead-ends metabolites allowed us to identify 448 biochemical reactions, with biological and/or genomic evidence for potato, which refined 40 metabolic pathways totally or partially, including glycolysis/gluconeogenesis, pyruvate metabolism, glyoxylate and dicarboxylate metabolism (photorespiration module), oxidative phosphorylation, carbon fixation in photosynthetic organisms, amongst others (Additional file 1). Additionally, and based on previous reports, we manually reviewed complete or partial pathways that induced a defense response in the plant, such as those related to accumulation of salicylic acid (ubiquinone and other terpenoid-quinone biosynthesis) and jasmonic acid (alpha-linolenic acid metabolism) [41], as well as other pathways related to PAMP (pathogen associated molecular patterns) signaling cascades (Additional file 1). By implementing automatic-specific-organism gap filling and semiautomatic-specific-organism gap find and gap fill, we were able to include 508 reactions in the reconstruction. The manual refinement and semiautomatic processes of the reconstruction are summarized in Table 1.

**Table 1:** Summary of the manual and semi-automatic refinement connectivity reconstruction

Refinement	New reactions	New metabolites	New pathways	Complete and partially complete pathways
Manual	448	215	6	42
Automatic and Semiautomatic	508	324	x	x
Total	956	539	6	42

### General model properties

**Fig. 1:**
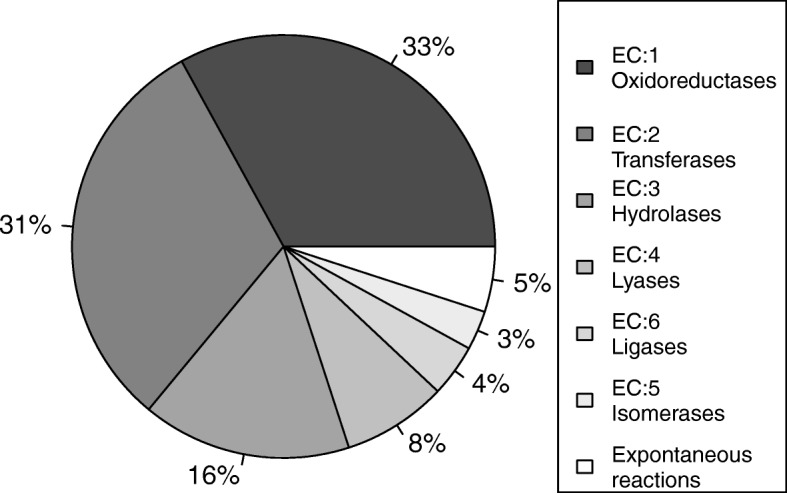
Required enzymatic activities to catalyze the reactions in the model

Hereby, we present a metabolic model of *S. tuberosum* in SBML format, hereafter denoted PstM1 (Additional file 2). This model accounts for 2765 genes, 1113 metabolic functions, 1773 GPR associations and 1938 metabolites involved in 87 central and peripheral metabolic pathways and 2072 reactions, of which 1254 could carry a non-zero flux given different objective functions and the specified biomass components. The required enzymatic activities (according to Enzyme Commission - EC) to catalyze the reactions in the model are shown in Fig. 1. Among the 2072 reactions, 2059 represent biochemical conversions and 13 represent exchange reactions with the environment, to describe the uptake/secretion of inorganic compounds (NO_3_, NO_2_^−^, Nitrile, CO_2_, O_2_, SO_4_^2−^, H_2_S, PO_4_^3−^, H_2_O, SO_4_^2−^, SeO_4_^2−^, NH_3_, Fe_2_^+^) and light (photon). FBA solutions showed that the model was able to simulate leaf biomass synthesis, which was represented by the conversions of biomass precursors: protein (amino acids), sugars, nucleotides, cell wall (cellulose, lignin precursors) and fatty acids (hexadecanoic acid).

### Comparisons with other C3 plant models

The model properties of PstM1 were compared with the versions of the genome-scale metabolic model of tomato (*Solanum lycopersicum*) [34], a closely-related species to potato, and other two C3 plant species, *Arabidopsis thaliana* [24, 25, 42] and rice (*Oryza sativa*) [33]. The PstM1 model contained 185 more reactions representing biochemical conversions than the initial model version of tomato. Furthermore, contained 666, 505 and 336 more total reactions than *A. thaliana* [24], *A. thaliana* [25] and rice [33] respectively. This difference is likely because we carried out a comprehensive refinement process and used different databases and resources to improve the connectivity of our metabolic reconstruction network. However, our model had 518 less reactions than the latest version of the *A. thaliana* model [42], which was updated from a comprehensively annotated database. In addition, our model had 71 fewer reactions than the total reactions set in the tomato model, where the additional reactions represent metabolite transporter processes. The PstM1 is not a compartmentalized model, since the main objective of this work was to evaluate the metabolic mechanisms associated to a possible decrease in photosynthetic capacity. Therefore, we focused on biochemical conversion reactions, such as light reactions and the Calvin cycle, which are catalyzed in only one more compartment than the intracellular transport process.

Our potato model contained fewer blocked reactions than the other C3 plant models compared here. However, the percentage of the reactions used for biomass synthesis in all these models was similar. The percentage for the potato model was 60.5%, compared to 56.5% for the tomato model, 67.3% for the *A. thaliana* model [25], and 57.3% for the rice model. During the time course of infection with *P. infestans* that were evaluated in this work, a total of 995 (48%) reactions in the potato model simulations could carry flux from nutrients to the specified biomass components, and in the simulations of the tomato model, between 16.8 and 17.3% of the reactions carried flux to represent three specific metabolic scenarios. It is probable that the reason for a higher number of reactions used in the potato model simulations is that the objective function was the maximization of leaf biomass synthesis, with the purpose of evaluating the possible metabolic scenarios in three moments (0, 1 and 3 days post inoculation) of the compatible interaction between potato and *P. infestans*. While in the tomato model, the objective function was the total flux minimization for three metabolic scenarios: heterotrophy, phototrophy and photorespiration.

### Metabolic phenotype analysis of compatible interaction between *S. tuberosum* and *P. infestans*

**Fig. 2:**
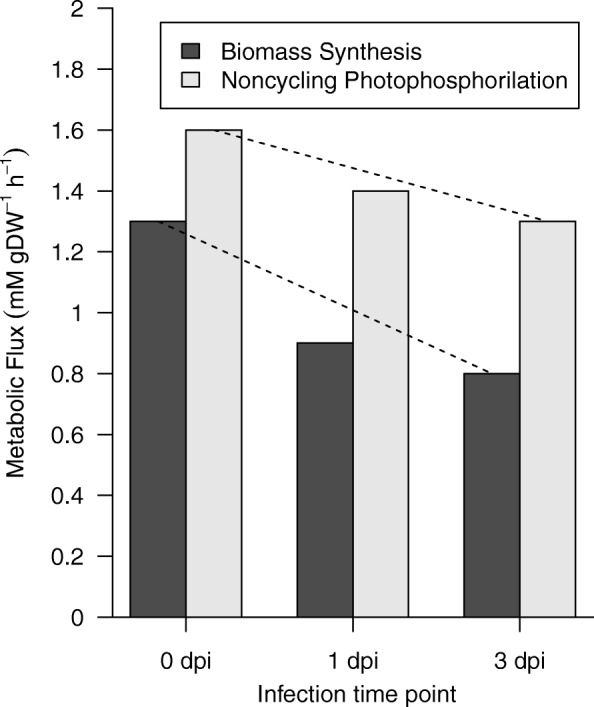
Metabolic fluxes of the biomass synthesis and noncyclic photophosphorylation reactions during compatible interaction between *S. tuberosum* and *P. infestans*. The flux reaction of biomass synthesis and noncyclic photophosphorylation decrease at 1 and 3 dpi

To determine the metabolic profile for each infection time point, we performed a FBA with flux boundaries established from the gene expression data (see Methods). Our study evidenced a decrease in the objective function (biomass synthesis of leaf) during the compatible interaction: 28% 1 day post inoculation (dpi) and 33% at 3 dpi, from the initial time 0 dpi (Fig. 2). These model predictions can be understood if we take into account that for the first interaction days, the pathogen grows well intercellularly and then intracellularly [16, 43] mainly in the leaves [44]. We hypothesize that this pathogenic invasion causes loss of metabolic reactions capacity of the primary metabolism to synthesize macromolecules in the leaf [45–47].

### Photosynthesis and carbon fixation metabolism

Photophosphorylation, the synthesis of ATP in photosynthesis, occurs in chloroplasts [48]. Noncyclic photophosphorylation requires photosystem I and photosystem II, and involves water oxidation, oxygen evolution and reduction of an acceptor. In contrast, cyclic photophosphorylation is driven only by photosystem I and works with light of wavelengths over 680 nm, and does not require water oxidation and oxygen evolution [49]. *S. tuberosum* PstM1 includes the definition of the following balanced photosynthetic light reactions taken from literature sources [33, 50, 51]:

1 $$ 7\ \mathrm{Photon}\left[\mathrm{c}\right]+3\ \mathrm{ADP}\left[\mathrm{c}\right]+3\ \mathrm{Orthophosphate}\left[\mathrm{c}\right]=>3\ \mathrm{ATP}\left[\mathrm{c}\right] $$

2 $$ 28\ \mathrm{Photon}\left[\mathrm{c}\right]+9\ \mathrm{ADP}\left[\mathrm{c}\right]+7\ \mathrm{NADP}+\left[\mathrm{c}\right]+9\ \mathrm{Orthophosphate}\left[\mathrm{c}\right]+9\ \mathrm{H}2\mathrm{O}\left[\mathrm{c}\right]=>7\ \mathrm{Oxygen}\left[\mathrm{c}\right]+7\ \mathrm{H}+\left[\mathrm{c}\right]+7\ \mathrm{NADP}\mathrm{H}\left[\mathrm{c}\right]+9\ \mathrm{ATP}\left[\mathrm{c}\right] $$

Our results showed that the metabolic flux for noncyclic photophosphorylation slightly diminished throughout the infection time points (Fig. 2) and that the flux for the cyclic photophosphorylation reaction was consistently zero. In this steady-state model we assumed that at 0 dpi the metabolic flux in the noncyclic photophosphorylation reaction synthetized enough ATP and NAPH for CO_2_ fixation; therefore, the metabolic flux in the cyclic photophosphorylation was not required [52]. The cyclic photophosphorylation reaction supplies only ATP [53] and its metabolic flux can be zero, because this reaction requires a balance in its input and output of electrons. Electron transport is zero when its components are completely reduced [48]. Our results suggest that during the interaction of the plant with the pathogen, there can be a reduction in the metabolic capacity of noncyclic photophosphorylation, and cyclic photophosphorylation could not supply the ATP deficit. These light reactions in a healthy plant convert light energy into chemical energy that is used for many cellular reactions that contribute to biomass synthesis [54, 55]. The low capacity in light reactions observed here could affect the flux of precursor reactions of biomass and, consequently, affect the capacity to synthesize biomass in the model. However, the flux of noncyclic photophosphorylation decreased less than biomass synthesis, which could be explained by the reduction of energy from photophosphorylation that is consumed by maintenance reactions different from the precursor reactions of biomass. Some of these maintenance reactions are associated with growth, for example, to maintain the electrochemical gradients across the plasma membrane, whereas others are independent of the specific growth rate of the cells [55].

**Fig. 3:**
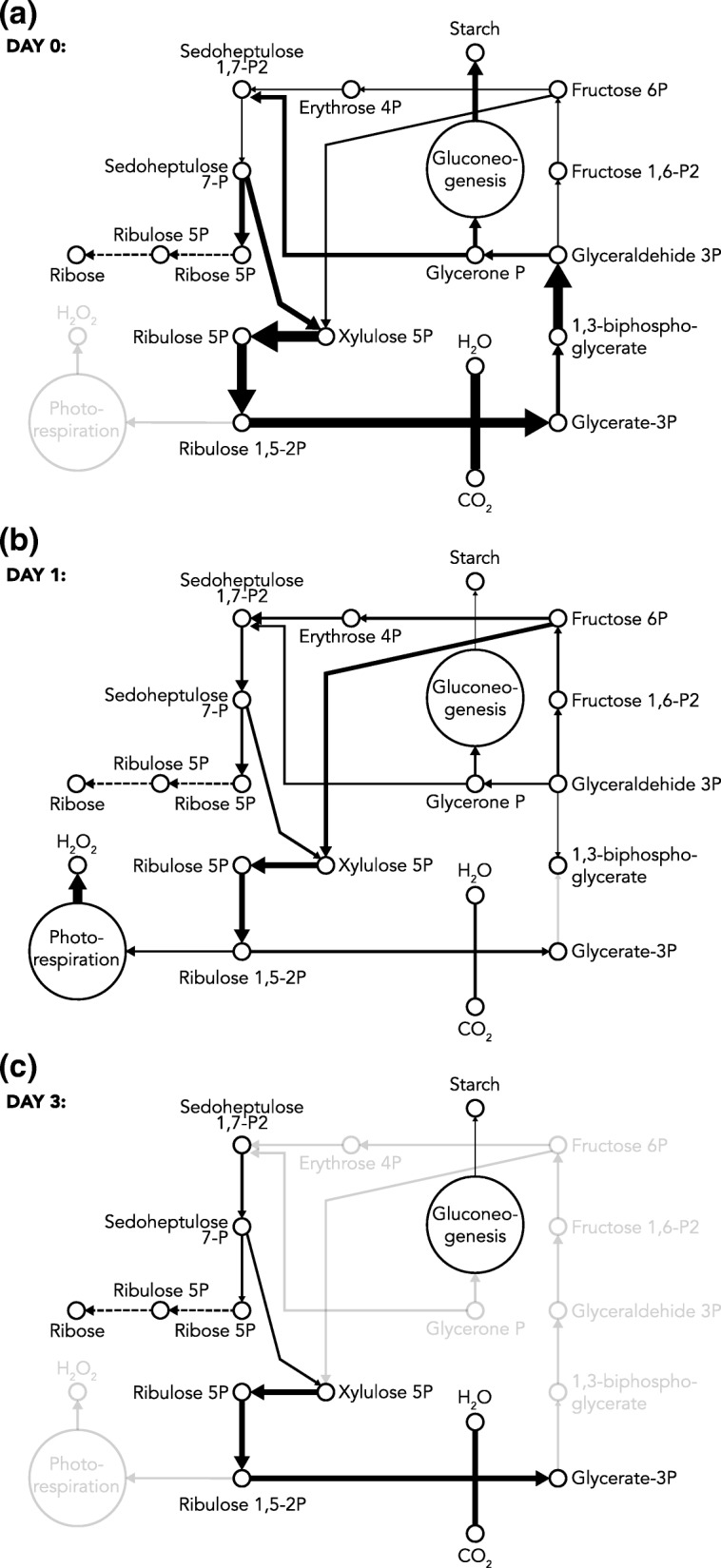
Metabolic flux distribution patterns of the fixation carbon pathway and some reactions associated with photorespiration and carbohydrate synthesis. **a** Metabolic flux distribution patterns for 0 dpi; **b** metabolic flux distribution patterns for 1 dpi; and **c** metabolic flux distribution patterns for 3 dpi

The decrease in photosynthetic ability during compatible interaction between *P. infestans* and *S. tuberosum* has been reported in other studies. For example, in a previous study, the decline in the efficiency of photosystem II [56] and downregulation of genes encoding proteins involved in photosynthesis was shown [16]. During the optimization of a metabolic network in steady state, we are able to predict the reaction flux, which is the overall rate of metabolite conversion [57]. We evaluated the fluxes distribution of the fixation carbon pathway to evaluate the photosynthetic capacity of the plant during all infection time points. The corresponding metabolic flux distribution patterns are shown in Fig. 3. A table listing the simulated flux values for each infection time point is given in Additional file 3. In general, the metabolic fluxes in the carbon fixation cycle (also known as the Calvin cycle) during potato late blight was characterized by the decrease and loss of reaction fluxes necessary to convert the inorganic carbon in organic carbon (Fig. 3). At 0 dpi, the metabolic fluxes of the reactions in the fixation carbon cycle varied between 5.79 and 51.42 mmol g ^− 1^ DW h^− 1^, suggesting interconversion of metabolites in all reactions in this pathway. At 1 and 3 dpi, the variation of the metabolic fluxes in response to the compatible interaction was from 0 to 28 mmol g ^− 1^ DW h^− 1^and from 0 to 25.7 mmol g ^− 1^ DW h^− 1^, respectively. At 3 dpi, six reactions completely lost their flux capacity, showing a higher decrease of the global metabolic capacity in this time compared with the two previous times of infection.

The Calvin cycle involves three main phases: 1) carboxylation of ribulose-1,5-bisphosphate (RuBP) to form 3-phosphoglycerate (PGA), mediated by Ribulose-1,5-bisphosphate carboxylase/oxigenase enzyme (RuBisCO); 2) reduction of PGA to the level of a sugar (CH_2_O) by formation of glyceraldehyde 3-phosphate (GAP) using NADPH an ATP produced in the light reactions; and 3) the regeneration of RuBP [72]. Hereafter, these stages are described for the three times of infection and the subsequent changes in the metabolic behavior are specified.

The highest flux of carboxylation of RuBP was obtained in the model optimization of 0 dpi (Fig. 3a). At this time, we observed that the RuBisCO enzyme had the highest carboxylase activity. In contrast, at 1 dpi, this enzymatic activity decreased, but increased the oxygenase activity of the RuBisCO, reducing the energy efficiency of photosynthesis in the plant [58, 59]. The subsequent metabolism of glycolate produced by the oxygenation of the RuBP is known as photorespiration, and is associated with high light intensity, uptake of O_2_ and increased H_2_O_2_ production [60, 61]. In the solution of this metabolic model at 1 dpi the uptake of photons is not enhanced, but H_2_O_2_ production is increased; the H_2_O_2_ is one of the major and the most stable reactive oxygen species (ROS) that regulates basic acclimation, developmental and defense processes in plants [62], including programmed cell death (PCD) [63]. One of the most rapid defense responses against pathogen attack is the oxidative burst, which consists of the production of ROS, primarily superoxide and H_2_O_2_, at the site of attempted invasion [64]. The quick generation of H_2_O_2_ in potato tuber tissue following inoculation with *P. infestans* was previously reported [65]. Our results suggest that the increase in photorespiration compared to the decrease in carboxylation of RuBP could be associated with the plant’s requirement to trigger a quick defense mechanism, given that photorespiration would appear to be the fastest process for generating H_2_O_2_ [66].

At 3 dpi, the oxygenation of the RuBP flux reaction disappeared, allowing an increase in the carboxylase activity of the RuBisCO. However, the flux of the carboxylation reaction was reduced by 55% compared to 0 dpi. Despite of the defense response through the oxidative burst observed in the previous time, the metabolic flux to H_2_O_2_ production here was zero. During incompatible plant-pathogen interaction, an initial and very rapid accumulation of H_2_O_2_ is followed by a second and prolonged burst of H_2_O_2_ production [67]. In addition, the activity and levels of the ROS detoxifying enzymes are suppressed by salicylic acid (SA) [68]. The suppression of ROS detoxifying mechanisms is crucial for the induction of PCD [69, 70], which potentially limits the spread of disease [67]. In our model simulation, only one peak of H_2_O_2_ production occurred (Fig. 3b). Interestingly, this behavior has been previously reported during compatible plant-pathogen interactions [71]. In addition, the metabolic flux that represents the synthesis of SA in PstM1 is consistently zero at the time points of infection evaluated. This can indicate that ROS-scavenging mechanisms are not downregulated in the metabolic model. Thus, just one peak of oxidative burst observed at 1 dpi is not sufficient to trigger an effective defense response in the plant; first, because a prolonged production of H_2_O_2_ is required, and second, because ROS production without suppression of ROS detoxification does not result in the induction of PCD [69, 70].

**Fig. 4:**
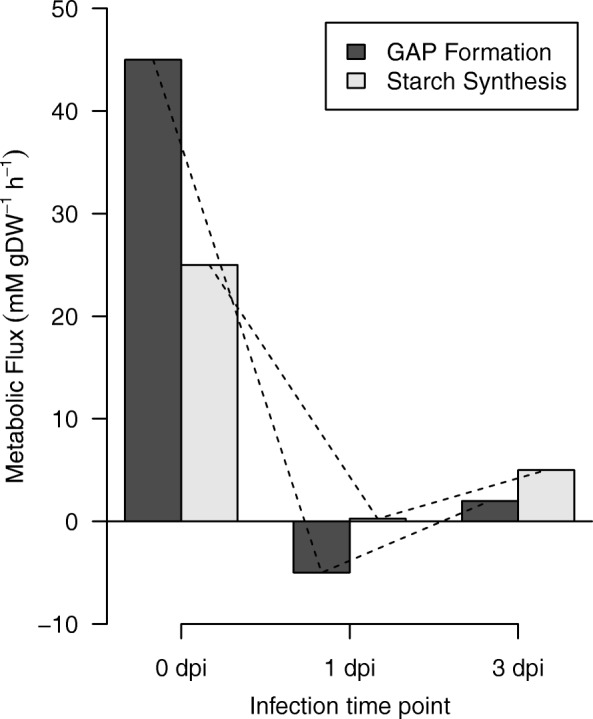
Metabolic fluxes of GAP formation and starch synthesis. The fluxes of these reactions showed the same trend through infection time point

In the reaction of GAP formation, the fluxes fall deeply from 0 to 1 dpi (Fig. 3), where the anabolic capacity of this reaction is null. At 3 dpi, the metabolic flux increased incipiently, likely due to the recovery of carboxylation of the RuBisCO. The GAP molecules can be used for the synthesis of sucrose or starch, and alternatively can be used to regenerate RuBP [72, 73]. We compared the fluxes of the starch synthesis reaction with the fluxes of PGA transformation in GAP through all time points of infection, based on the idea that starch production is a good indicator of a healthy plant, which will only store extra reservoirs of starch in tissues if all their energy requirements are already fulfilled [74]. In addition, depending on the developmental stage of the plant, the starch has been identified as an important integrator in plant growth regulation to cope with continual changes in carbon availability when the rate of photosynthesis is modified by environmental constraints [74, 75]. The comparison between these two reactions showed the same change trend in metabolic fluxes (Fig. 4). At 1 dpi, the metabolic flux to synthesize GAP and starch was the lowest, and at 3 dpi, the flux of this reaction increased incipiently. Our results indicated that the loss of carbon fixation capacity of the RuBisCO and the subsequent decrease in GAP formation from PGA, can be related to the decreased efficiency of starch biosynthesis.

Previous studies have shown that starch biosynthesis regulation is linked with the expression of ADP-Glc pyrophosphorylase (AGPase) in leaves [75, 76]; likewise, AGPase activity is generally activated by PGA [77]. Therefore, AGPase activation or inactivation by PGA production allows starch synthesis to be adjusted in response to changes in photosynthesis [78]. Our model suggests that the metabolic flux of the AGPase activity decreased slightly through infection time points (view Additional file 3). This can be associated with the general decline in the efficiency of starch synthesis in response to the decrease of biosynthesis of GAP from PGA. Although the AGPase activity and starch biosynthesis both tend to decrease, they show particular tendencies that are not comparable between them, especially from 1 to 3 dpi (view Additional file 3). Hence, additional regulatory mechanisms could be required to achieve changes in the rate of starch biosynthesis, as previously reported [75, 79–82].

In the Calvin cycle, the RuBP is both consumed and synthesized [83]. The synthesis of RuBP is known as regeneration, and involves a series of reactions from GAP, that are energetically favorable and do not consume ATP or NADPH, except in the phosphorylation reaction to transform ribulose 5-phosphate into RuBP [84]. The flux distribution of these reactions was affected during compatible interaction (Fig. 4) mainly at 3 dpi, where a null flux is obtained for three reactions. The net flux of transformation of ribulose 5-phosphate in RuBP reaction decreased approximately by 55% at 1 and 3 dpi compared to 0 dpi. This can be a consequence of both, the decrease in the metabolic flux of its precursor’s reactions in the cycle as well as the decrease of the metabolic capacity of ATP production for the light reactions. In addition, we observed that at 0 and 3 dpi the metabolic fluxes of the regeneration and carboxylation of RuBP are directly proportional. At 1 dpi, the metabolic flux of the regeneration was directly proportional to the sum of the flux for carboxylation and oxygenation. Overall, these 7results can indicate that in our model the RuBP is synthesized in the same rate that it is consumed. This observation agrees with previous studies that demonstrated that, in a photosynthetic steady state model, the rate at which RuBisCO consumes RuBP equals the rate at which RuBP is regenerated [85, 86].

## Conclusions

In this work, we simultaneously introduced the first metabolic network of *S. tuberosum* and the first genome-scale metabolic model of the compatible interaction of a plant with *P. infestans*. The metabolic flux of the light reactions and carbon fixation cycle, including photorespiration and starch synthesis, suggests a suppression of the photosynthetic capacity as consequence of the compatible interaction between *P. infestans* and *S. tuberosum*. Our results suggest that the suppression of the photosynthetic capacity could be associated with a quick defense mechanism, which is not sufficient to trigger an effective defense response in the plant. The results shown here are in silico metabolic predictions, which closely match previous studies of plant physiology.

The PstM1 generated in this study can be used to simulate different metabolic scenarios of the potato plant, integrating gene expression data through constraints into fluxes of the reactions. For its part, the genome-scale metabolic model of the compatible interaction of potato plant allows the prediction of other metabolic mechanism involved during patosystem activity and is an useful tool for proposing novel directed hypotheses, guiding research to conduct metabolic engineering in the plant, and identifying emerging properties of the compatible interaction, which otherwise could not be observed through the study of individual molecules and processes. Moreover, PstM1 reconstruction alone can also be informed by new biological data in order to highlight different processes relative to potato metabolism.

## Methods

### Metabolic reconstruction

An initial automatic draft reconstruction was first created by means of RAVEN [87] software toolbox based on the PGSC potato genome sequence [88]. Both automatic reconstructions were then conciliated and merged through in-house scripting. This merged reconstruction was taken as a starting point for manual reconstruction and then subjected to a manual refinement based on literature and available biological data.

### Metabolic reconstruction refinement

**Fig. 5:**
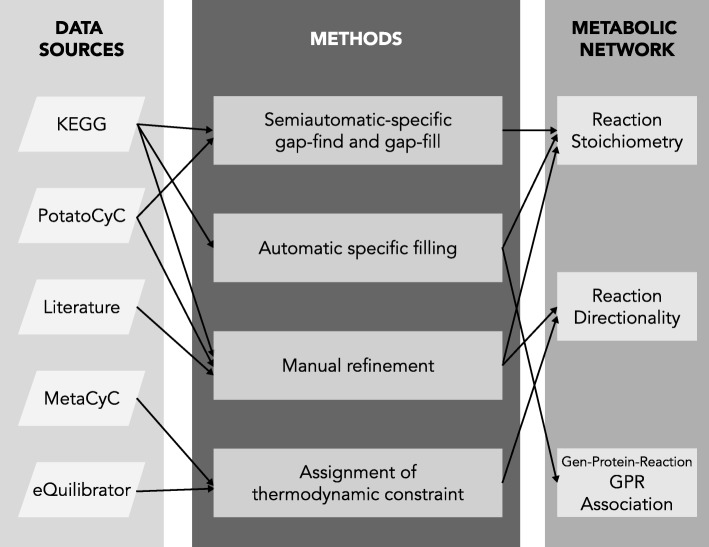
General scheme of the refinement of potato metabolic network. The lines indicate the trajectory from data sources to the refined metabolic network

The reconstruction refinement stage was divided into six phases: (1) manual gap refinement of the metabolic network and manual refinement of the reversibility and directionality of the reactions; (2) automatic-specific-organism filling; (3) semiautomatic-specific-organism gap find and gap fill; (4) establishment of the directionality and reversibility of the reactions through Gibbs free energy of reaction value (ΔrG′°) [89]; (5) inclusion of exchange reactions; and finally (6) definition of gene-protein-reaction associations (Fig. 5).

During manual refinement, the network’s connectivity was verified in a pathway by pathway basis, as well as in a metabolite by metabolite basis [35]. For this, a search for dead-end metabolites in the reconstruction was performed by means of the R package minval [90]; these metabolites correspond to those that are consumed in the set of biochemical reactions, but are not synthesized and vice versa. Based on the identified dead-end metabolites, missing reactions in the reconstruction were manually tracked within the KEGG Pathway Maps database [91, 92], which associates organism-specific genomic information to metabolic pathways maps. For all those reactions not reported for *S. tuberosum* in the KEGG Pathways database, we verified that their catalyzing enzymatic activity was reported in the Plant Metabolic Network (PMN) database specific for potato (PotatoCyc) [93]. In addition, based on biochemical literature, carbohydrate and energetic metabolic pathways were further refined. During the entire manual review process of the network’s connectivity, reactions without any type of biological and/or genomic evidence for this plant were removed, and the reversibility and directionality of 120 reactions were corrected.

In order to verify that the metabolic pathways reported in this reconstruction were present in the metabolism of *S. tuberosum*, we used the KEGGREST R package, which allowed us to obtain all of the metabolic pathways reported for this organism in the KEGG pathways database [94]. By comparing the metabolic pathways obtained from KEGG with those in the reconstruction, we found 12 non-reported pathways, which were then removed. Since some of the reactions belonging to these pathways were catalyzed by enzymatic activities reported in PotatoCyc, these were included in the reconstruction without being associated to any specific metabolic pathway.

During the process of automatic-specific-organism filling, a database construction of all the biochemical reactions reported for *S. tuberosum* in KEGG was done using the g2f R package [95]. Later, only missing reactions were added to the reconstruction, using the sot_g2f in-house script [96]. Since the network still showed gaps, a semiautomatic-specific-organism gap finding and gap filling process was performed using the g2f package. For this, once again we identified all of the dead-end metabolites in the network and automatically tracked them to a reference database that contains all of the biochemical reactions stored in KEGG. The gap fill search method was restricted to retrieve only reactions that showed metabolites in the reconstruction. Finally, through manual validation, we included only reactions with enzymatic activities reported for *S. tuberosum* in PotatoCyc.

The reversibility and directionality of the reactions that were not manually corrected were determined through Δ_*r*_*G′°* values obtained from EQuilibrator [97] and MetaCyc [98] databases. These databases establish the value for Δ_*r*_*G′°* by calculating the Gibbs free energy of formation of a compound (Δ_*f*_*G*′°), through the group contribution method for thermodynamic analysis [89]. The parameters used to calculate Δ_*f*_*G*′° in Equilibrator are pH 7.0 and ionic strength 0.1, and in MetaCyc these are pH 7.3 and ionic strength 0.25. Since the parameters are located in a pH range of 7.0–7.3 and ionic strength of 0.1–0.25, the reversibility R script [99] was implemented, which allowed us to compare the Δ*rG*′° values from both databases. When both reversibilities were below − 1, the GEMR reversibilities were established as forward irreversible. Due to lack of biological evidence and discrepancies between the Δ*rG*′° values of the databases, the remaining reactions were determined as reversible.

During the fifth phase of the reconstruction refinement process, we included 13 exchange reactions which transport metabolites that cannot by synthesized by the cell, from the limits of the system to the cytoplasm, that are precursors to other metabolites [24]. Finally, once the reconstruction was refined in terms of its reactions, metabolic pathways and reversibility, GPR associations were integrated using the g2f package. This package constructs GPRs based on KEGG ORTHOLOGY of the reactions present in the reconstruction.

### Transformation of reconstructed network into a genome scale metabolic model

From the genome-scale metabolic reconstruction of potato, a SBML version was generated using the Python package COBRApy [100]. Consecutively, the SBML file was imported to the R package Sybil [101], where the whole set of reactions present in metabolic reconstruction was transformed into a steady state metabolic model *S*_*ij*_ *v*_*j*_ = *0*. Where S_*ij*_ is the entries of the stoichiometric matrix (Additional file 4). Rows in S represent metabolites and columns represent reactions, and *v*_*j*_ is the metabolic flux vector for each reaction. Substrates in the reaction have negative coefficients, while products have positive ones. This matrix also takes into account transport reactions across the cell membrane, which are represented as reactions interconverting intracellular and extracellular compounds. In the metabolic model, the reaction fluxes are subjected to constrains (*− v*_*jmin*_ *≤ v ≤ v*_*jmax*_) [25, 27].

Objective function was defined by the biomass synthesis reaction in the leaf *OF*_*bioamass*_ (Additional file 4), previously reported in the genome-scale metabolic model of *Arabidopsis thaliana* [25]. This objective function was mathematically written as a combination of metabolic coefficients of the biomass composition estimated for slow-growing species [102, 103].

### Incorporation of gene expression data into the genome-scale metabolic model

Gene expression data of the compatible interactions was obtained from the study of Gyetvai et al. [17]. We used the normalized libraries of the untransformed cultivar Désireé at three infection time points with *P. infestans* (zero (0 dpi), one (1 dpi) and three (3 dpi) days post inoculation), with three biological replicates. The tag annotation was performed by BLASTN [104]. The source for the tag annotation was the available RNA sequence of “Potato 3.0” [105]. The unique genes and maximum value counts per gene were performed with the script summarization.r [106]. As the last step for building the gene expression database, all refseq gene identifiers were transformed to Entrez identifiers, by means of the R package UniProt.ws [107].

From the previously built gene expression database, we generated an expression set for each infection time point by means of the R package Biobase [108]. The gene expression values were incorporated directly into fluxes constraints of the reactions in the model using the R package ex2flux [109], which implements a method to integrate gene expression values into each GPR associated to the reactions of the model.

### Metabolic flux model optimization

FBA represents a constraint-based modeling approach that allows the prediction of metabolic steady-state fluxes, by applying mass balance constraints into a stoichiometric model [38]. The reactions in the model can be represented by a linear system of equations, then, problems such as maximizing specific chemical production or growth can be solved by linear programming [21, 110]. With the purpose of obtaining a computational distribution of metabolic fluxes, FBA was employed assuming maximization of the objective function, by means of the R package Sybil (Eq. 3) maximization *OF*_*bioamass*_

subject to

3 $$ {\displaystyle \begin{array}{c}\sum {S}_{ij}\;{v}_j= 0\\ {}\mathrm{i}=1,2\dots \mathrm{m}\kern0.5em \mathrm{j}=1,2\dots \mathrm{n}\\ {}-{v}_{jmin}\le v\le {v}_{jmax}\end{array}} $$

To constrain the space of all the possible steady-state flux distributions in the optimization, we imposed thermodynamic constraints to reaction reversibility as well as upper and lower bounds constraints on reactions fluxes from known expression values for the particular enzyme that catalyzes the reaction.

## Additional files

Additional file 1: Refined pathways in the metabolic network reconstruction. (PDF 88 kb)

Additional file 2: Genome scale metabolic model of *S. tuberosum* in SBML format. (XML 2677 kb)

Additional file 3: Metabolic fluxes of the carbon fixation pathway and other reactions of interest. (PDF 34 kb)

Additional file 4: Genome scale metabolic network reconstruction of *S. tuberosum* in xls format. (XLSX 161 kb)

## Abbreviations

AGPase: ADP-Glc pyrophosphorylase
EC: Enzymatic activities (according to Enzyme Commission)
FBA: Flux balance analysis
GAP: Glyceraldehyde 3-phosphate
GEMR: Genomic-scale metabolic reconstructions
GPR: Gene-protein-reaction
PCD: Programmed cell death
PGA: 3-phosphoglycerate
ROS: Reactive oxygen species
RuBisCO: Ribulose-1,5-bisphosphate carboxylase/oxigenase enzyme
RuBP: Ribulose-1,5-bisphosphate
SA: Salicylic acid
