# Supplementary material for: A genome-scale metabolic model of potato late blight suggests a photosynthesis suppression mechanism
Source: BMC Genomics. 2018 Dec 11;19(Suppl 8):863. doi: 10.1186/s12864-018-5192-x (PMC6288859; doi:10.1186/s12864-018-5192-x)
Supplement: Supplementary file 1 — Refined pathways in the metabolic network reconstruction. (PDF 88 kb) [file 12864_2018_5192_MOESM1_ESM.pdf]

Supplementary Table S1.

Refined pathways in the metabolic network reconstruction. We show all the pathways included in the metabolic network of *S. tuberosum*. Pathway association was assigned based on the categorization of the KEGG Pathways database. The colors correspond to the level of curation of each pathway

Complete	Partially complete	Intervened	Not intervened

Alpha-Linolenic acid metabolism	Arginine and proline metabolism	Ascorbate and aldarate metabolism	Carbon fixation in photosynthetic organisms	Citrate cycle (TCA cycle)	Flavonoid biosynthesis	Galactose metabolism	Inositol phosphate metabolism
Nitrogen metabolism	Oxidative phosphorylation	Photosynthesis (ligth reactions)	Porphyrin and chlorophyll metabolism	Pyruvate metabolism	Steroid biosynthesis	Sulfur metabolism	Alanine, aspartate and glutamate metabolism
Benzoxazinoid biosynthesis	Brassinosteroid biosynthesis	Carotenoid biosynthesis	Cyanoamino acid metabolism	Cysteine and methionine metabolism	Diterpenoid biosynthesis	Fructose and mannose metabolism	Glutathione metabolism
Glycerolipid metabolism	Glycerophosph olipid metabolism	Glycolysis / Gluconeogenes is	Glyoxylate and dicarboxylate metabolism	Histidine metabolism	Lysine biosynthesis	Lysine degradation	Other types of O-glycan biosynthesis
Pantothenate and CoA biosynthesis	Propanoate metabolism	Selenocompo- und metabolism	Sphingolipid metabolism	Starch and sucrose metabolism	Terpenoid backbone biosynthesis	Ubiquinone and other terpenoid- quinone biosynthesis	Zeatin biosynthesis
Thiamine metabolism	Amino sugar and nucleotide sugar metabolism	Biosynthesis of unsaturated fatty acids	Butanoate metabolism	Caffeine metabolism	Carbon metabolism	Fatty acid elongation	Fatty acid metabolism
Glycine, serine and threonine metabolism	Linoleic acid metabolism	Monoterpenoid biosynthesis	Pentose phosphate pathway	Phenylalanine metabolism	Phenylalanine, tyrosine and tryptophan biosynthesis	Phenylpropano id biosynthesis	Purine metabolism
Pyrimidine metabolism	Tryptophan metabolism	Valine, leucine and isoleucine biosynthesis	Aminoacyl- tRNA biosynthesis	Anthocyanin biosynthesis	Arachidonic acid metabolism	Arginine biosynthesis	beta-Alanine metabolism
Biotin metabolism	Ether lipid metabolism	Fatty acid biosynthesis	Flavone and flavonol biosynthesis	Folate biosynthesis	Glucosinolate biosynthesis	Glycosaminogl ycan degradation	Glycosphingoli- pid biosynthesis - globo series
Glycosylphosph atidylinositol (GPI)-anchor biosynthesis	Lipoic acid metabolism	Limonene and pinene degradation	N-Glycan biosynthesis	Nicotinate and nicotinamide metabolism	One carbon pool by folate	Pentose and glucuronate interconversio ns	Riboflavin metabolism
Stilbenoid, diarylheptanoid and gingerol biosynthesis	Synthesis and degradation of ketone bodies	Taurine and hypotaurine metabolism	Tropane, piperidine and pyridine alkaloid biosynthesis	Tyrosine metabolism	Valine, leucine and isoleucine degradation	Vitamin B6 metabolism	
