# Supplementary material for: A genome-scale metabolic model of potato late blight suggests a photosynthesis suppression mechanism
Source: BMC Genomics. 2018 Dec 11;19(Suppl 8):863. doi: 10.1186/s12864-018-5192-x (PMC6288859; doi:10.1186/s12864-018-5192-x)
Supplement: Supplementary file 3 — Metabolic fluxes of the carbon fixation pathway and other reactions of interest. (PDF 34 kb) [file 12864_2018_5192_MOESM3_ESM.pdf]

Supplementary Table S2

Reaction fluxes of the metabolic pathways of interest in *S. tuberosum* to evaluate photosynthetic capacity through compatible interaction with *P. infestans.*

Pathways	Reaction (ID KEGG)	Metabolic flux (0 dpi)	Metabolic flux (2 dpi)	Metabolic flux (3 dpi)
**Biomass Synthesis**	RBS01	1,313856	0,9435836	0,8790459
**Cyclic Photophosphorylation**	RK0004	0	0	0
**Non Cyclic Photophosphorylation**	RK0005	1,630357	1,408214	1,35
**Carbon Fixation**	R00024	46,38961	14,34539	25,7072
	R01512	19,59667	0	0
	R01061 *rev	-45,65	5,326667	-1,176667
	R01015	17,39	8,923333	0
	R01068 *rev	-5,79	-13,63667	0
	R00762	5,79667	13,63667	0
	R01067	5,79667	13,63667	0
	R01829	5,79667	12,67667	0
	R01845	5,79667	12,67667	13,94333
	R01641	25,52	12,91333	10,93469
	R01056	-5,036667	-2,386667	-2,251979
	R01523	46,3891	25,61669	25,7072
	R01529 *rev	-51,426	-28,00336	-25,65781
	R01051	-5,036667	-3.62	-5,69
**Photorespiration**	R03140	0	11,2713	0
	R01334	0	11,2713	0
	R00475	0	39,43	0
	R00009	0	36,37207	3,992193
	R00372	0	0	65,06771
	R00945	-40,9	2,556667	-2,646667
	R01221	28,57	10,43925	14,86955
	R00588	693,29	1543,01	854,7267
	R01388	-4,417	-3,146667	-19,57333
	R01514	0	0	0
**AGPase activity**	R00948	0.3481719	0.2500497	0.2329472
**Starch Synthesis**	R02110	26,29711	0,25	5,42
**Salicylic Acid Synthesis**	RK0003	0	0	0

* rev = The direction of the metabolic flux in the metabolic pathway is reversed
